# Three-dimensional assessment of airway volumes in patients with unilateral cleft lip and palate

**DOI:** 10.1186/s40510-021-00382-4

**Published:** 2021-11-08

**Authors:** Bita Kiaee, Ludovica Nucci, Farzin Sarkarat, Ahmad Reza Talaeipour, Sara Eslami, Faezeh Amiri, Abdolreza Jamilian

**Affiliations:** 1 Department of Orthodontic, Tehran University of Medical Sciences, Tehran, Iran; 2 Multidisciplinary Department of Medical-Surgical and Dental Specialties, University of Campania Luigi Vanvitelli, Naples, Italy; 3 Department of Oral and Maxillofacial Surgery, Dental School, Cranio Maxillofacial Research Center, Tehran Medical Sciences, Islamic Azad University, Tehran, Iran; 4 Department of Oral and Maxillofacial Radiology, Dental School, Cranio Maxillofacial Research Center, Tehran Medical Sciences, Islamic Azad University, Tehran, Iran; 5 Orthodontist at Private Orthodontic Office, Tiergartenstraße 130, Hannover, Germany; 6 DDS, Tehran University of Medical Sciences, Tehran, Iran; 7 Department of Orthodontics, Dental School, Cranio Maxillofacial Research Center, Tehran Medical Sciences, Islamic Azad University, Tehran, Iran

**Keywords:** Cleft lip and palate, Nasopharyngeal space, Pharynx, Airway volume, Cone-beam computed tomography

## Abstract

**Background:**

Considering the adverse consequences of respiratory insufficiency in cleft lip and palate (CLP) patients, this study aimed to assess the pharyngeal airway dimensions in 9–12-year-old patients with unilateral CLP. This historical cohort evaluated the cone-beam computed tomography (CBCT) scans of 30 patients with non-syndromic unilateral CLP between 9 and 12 years and 30 age- and sex-matched non-cleft controls. Three-dimensional (3D) images were reconstructed by the Mimics software, and the nasopharyngeal, oropharyngeal, and total airway volumes, as well as the minimal cross-sectional area of the airway (minAx), and posterior airway length (PAL) were all measured in the sagittal plane. Data were analyzed by the Student’s *t* test.

**Results:**

The oropharyngeal and the total airway volumes, as well as the minAx and PAL in CLP patients, were significantly smaller than the corresponding values in the control group (*P* < 0.05). Despite smaller nasopharyngeal airway volume in CLP patients than controls, this difference was not statistically significant (*P* > 0.05).

**Conclusions:**

Nine- to twelve-year-old non-syndromic unilateral CLP patients have smaller pharyngeal airway dimensions than non-cleft controls, and are therefore at higher risk of respiratory insufficiency.

## Background

Cleft lip and palate (CLP) are among the most common orofacial anomalies with a prevalence of 1–7 per 1000 live births [1, 2].

CLP patients usually suffer from respiratory problems such as mouth breathing, noisy breathing, snoring, and sleep hypopnea due to nasopharyngeal abnormalities (nasal septal deviation, nasal atresia, and alar constriction) [3–5]. Approximately 70% of CLP patients have nasal airway problems, out of which, 80% show some degrees of mouth breathing [6, 7]. Also, it has been demonstrated that decreased nasal airway volume increases the susceptibility to mouth breathing, sleep hypopnea, and speech problems such as hyponasality [8]. In addition to nasopharyngeal deficiency, CLP patients also suffer from the dysfunction of the muscles controlling the soft palate, which can increase the risk of sleep-disordered breathing in combination with structural discrepancies of the maxilla and mandible (such as the maxillary or mandibular retrognathism, short body of the mandible, and downward and backward rotation of the mandible [9–14]). Knowledge about the anatomy and physiology of the craniofacial structures, especially in patients with modified or abnormal growth (such as CLP), is highly important for orthodontic treatment planning.

To date, several studies have evaluated the airway volume in CLP patients using two-dimensional (2D) lateral cephalometry, which has low diagnostic accuracy for this purpose, despite its simplicity and low cost [15–19]. CBCT is a three-dimensional (3D) imaging modality with numerous dental applications, which has much higher accuracy than lateral cephalometry for this purpose at a comparable cost.

Controversy exists regarding the airway volume in CLP patients compared with non-cleft controls. Some studies reported significantly smaller pharyngeal airway volume in CLP patients [20–28], while some others demonstrated no significant difference or larger airway volumes in CLP patients than controls [10, 29–33]. Thus, this study aimed to assess the pharyngeal airway dimensions in non-syndromic unilateral CLP patients between 9 and 12 years in comparison with age- and sex-matched non-cleft controls using CBCT.

## Methods

This historical cohort evaluated the CBCT scans of 30 patients with non-syndromic unilateral CLP and 30 non-cleft controls between 9 and 12 years. The study protocol was approved by the ethics committee of the Department of Orthodontics, School of Dentistry (IR.IAU.DENTAL.REC.1395, 41). This study evaluated the available CBCT scans of non-syndromic unilateral CLP patients between 9 and 12 years who were in cervical vertebral stage (CVS) II and III and had undergone similar surgical procedures by the same surgeon for lip and hard tissue closure before the age of 3.5 years.

The exclusion criteria were history of previous orthodontic treatment, orthognathic surgery, trauma, syndromes, history of tonsillectomy/adenoidectomy, history of treatment with continuous positive airway pressure, history of medication intake, history of upper airway obstruction, history of frequent colds (more than 6 times in the past 1 year), and having a cold or upper airway inflammatory disease at the time of taking the CBCT scans. Thirty controls were also selected who had class I skeletal relationship (ANB angle between 0° and 4°), and normal growth pattern (SN-MP angle 32° ± 2°), and did not have any of the above-mentioned exclusion criteria. The CBCT scans had been taken for purposes not related to this study, such as assessment of an impacted tooth. The control subjects were matched with the CLP patients in terms of age, CVS, and gender. The CVS of the participants was determined based on their CBCT sagittal view, and the measurements were made by two experienced blinded observers.

All CBCT scans had been obtained in standard condition at maximum intercuspation and in an upright position by NewTom 5G CBCT scanner (QR, Verona, Italy). The scanning time was 14 to 18 s, the exposure time was 3.4 s, and the voxel size was 0.3 mm. The 3D data were reconstructed according to the method described by Celikoglu et al. [21]. Next, 3D images were reconstructed with 0.25 mm slice thickness in DICOM format and transferred to Materialise Mimics Innovation Suite 20 software (Leuven, Belgium). The images were reoriented in the software such that their horizontal reference plane was the Frankfurt plane (passing through the right and left porion and the right and left orbitale). The sagittal reference plane was drawn perpendicular to the horizontal plane, passing through the nasion and midorbital points. The axial plane was perpendicular to the previous two planes, passing through the nasion. After standardization of the images in terms of orientation, the pharyngeal airway was outlined as follows:

(I) Its anterior border was a plane that started from the intersection of the vertical plane drawn from the sella and the nasion–nasion plane and continued to the most inferior border of the vomer bone in the sagittal plane.
(II) Its posterior border was the posterior wall of the pharynx.
(III) Its inferior border was a plane tangent to the most caudal medial projection of the third cervical vertebra, perpendicular to the sagittal plane.
(IV) Its superior border was the nasal floor.

**Fig. 1:**
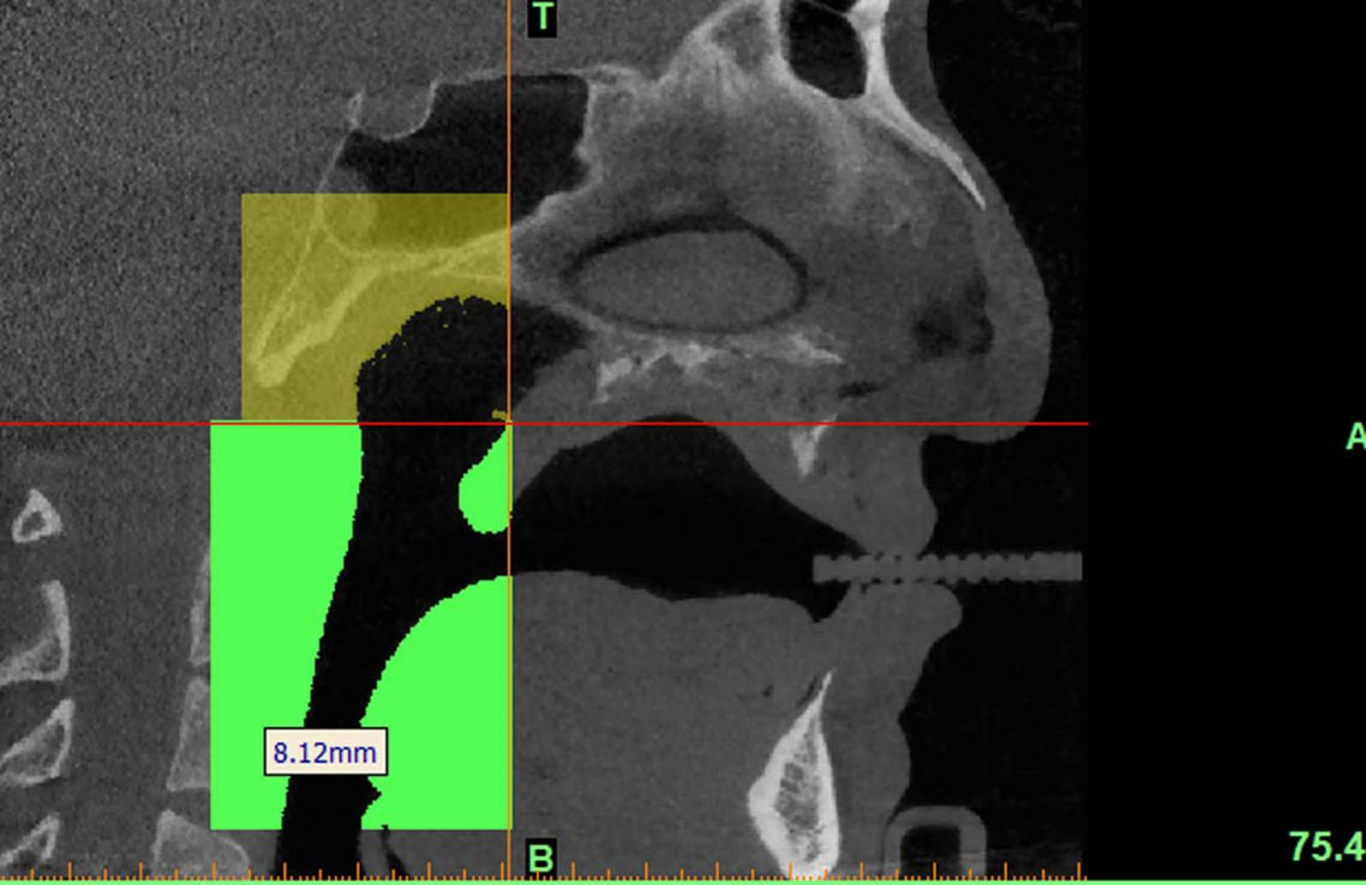
Outlining the pharyngeal airway

**Fig. 2:**
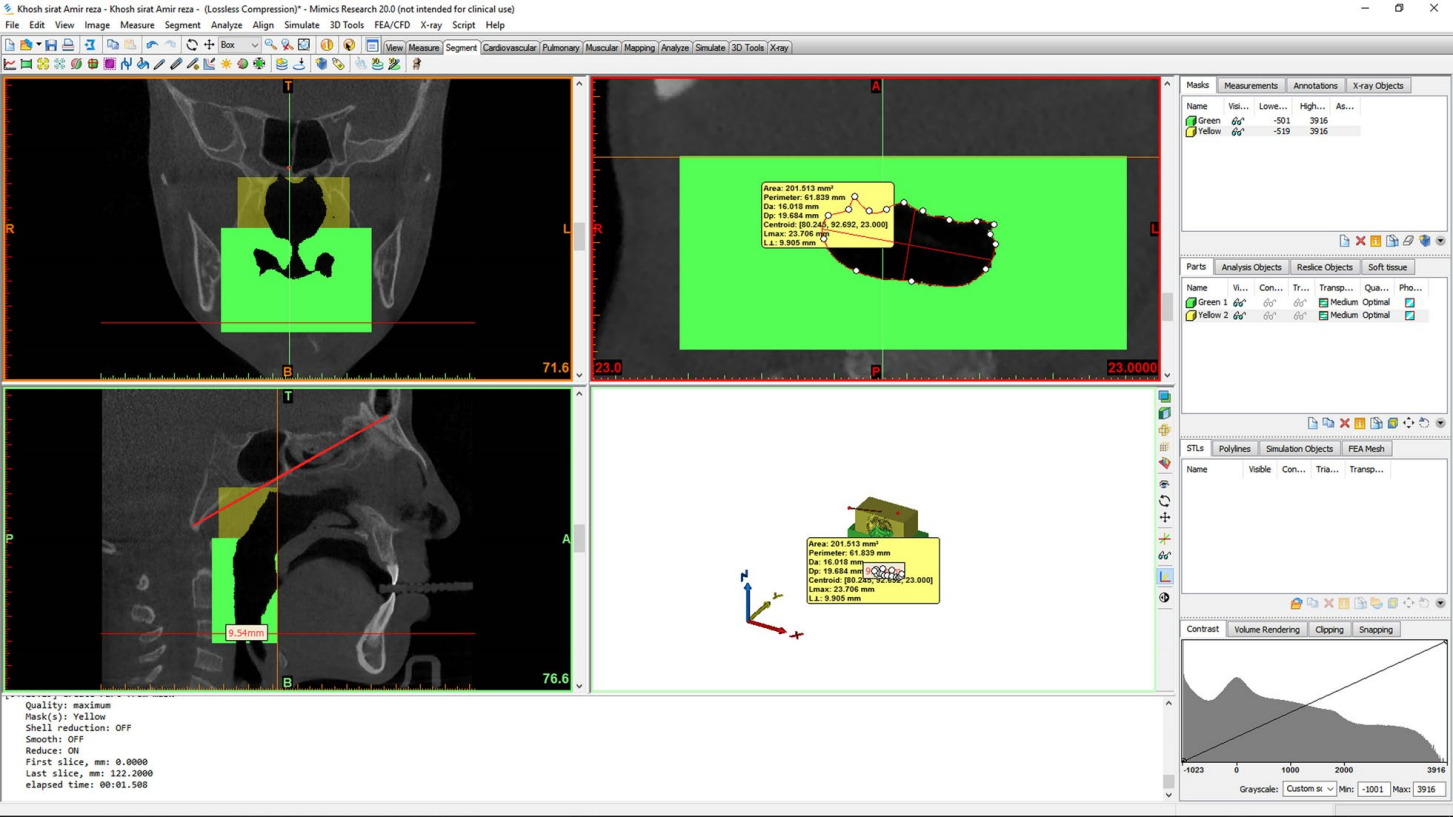
Measuring the airway cross-sectional area on axial sections

**Fig. 3:**
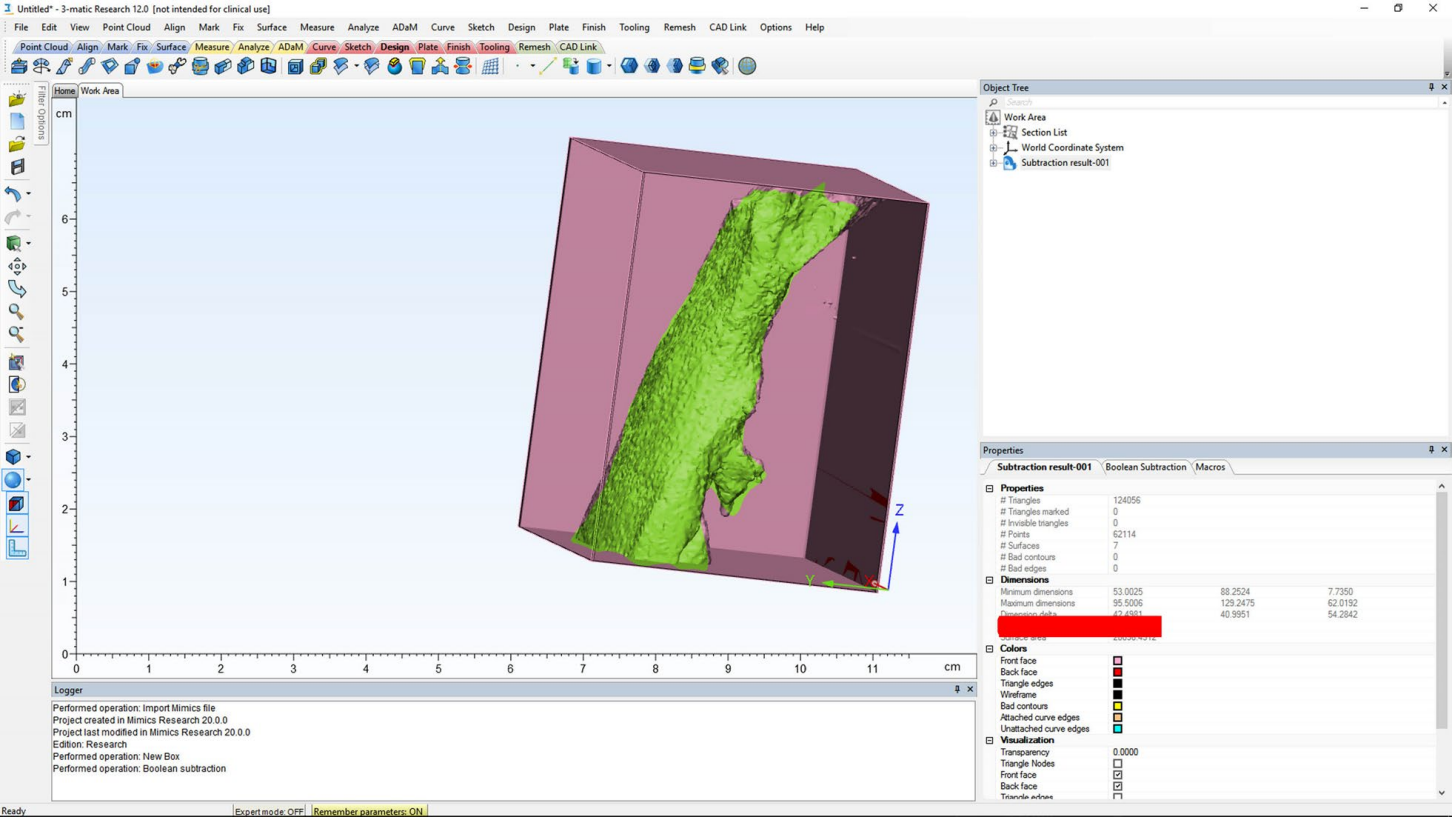
Measuring the oropharyngeal airway volume

**Fig. 4:**
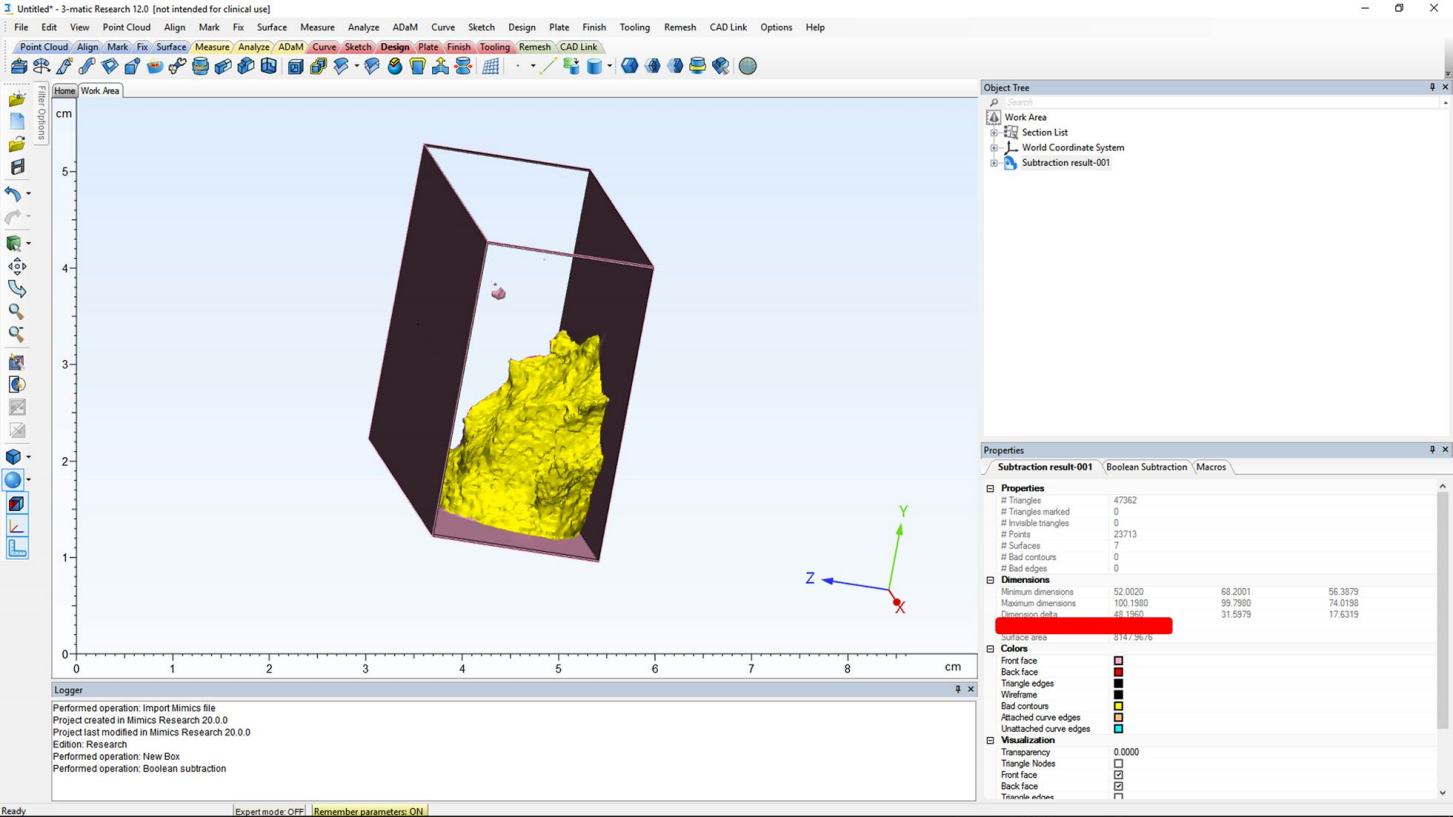
Measuring the nasopharyngeal airway volume

A plane perpendicular to the sagittal plane that passed through the most caudal superior part of the first cervical vertebra divided the pharyngeal airway into an upper (nasopharyngeal) and a lower (oropharyngeal) section (Fig. 1). The posterior airway length (PAL), which is the narrowest space posterior to the base of the tongue, was also measured as a line in the sagittal plane and reported in millimeters (mm). The cross-sectional area of the narrowest part at the base of the tongue (minAx), which was the minimal cross-sectional area of the PAL, was also measured on axial sections and reported in square millimeters (mm^2^) (Fig. 2). The three-dimensional measurements of the airway volumes were then performed by the Mimics software (Figs. 3, 4).

All measurements were made by two calibrated examiners. Ten images (approximately 30% of the data) were randomly selected and measured again by the same examiners (without access to the first measurements) 2 weeks after the first assessment in order to analyze the reliability of the measurements. The intra-examiner reliability was tested by the Pearson’s correlation coefficient. The intra-examiner reliability scores were found to be over 0.7 by test–retest reliability assessment. Thus, the results had substantial agreement.

Data were analyzed using SPSS version 22 (SPSS Inc., IL, USA). Normal distribution of data was evaluated by the Shapiro–Wilk test. Since data were normally distributed, the Student’s *t* test was used to compare the airway volumes and cross-sectional area between the patient and control groups. Level of significance was set at 0.05.

## Results

Of 30 patients with unilateral CLP, 14 (46%) were females and 16 (54%) were males, with a mean age of 10.3 ± 0.8 years. Of 30 controls, 14 (46%) were females and 16 (54%) were males, with a mean age of 10.9 ± 1.2 years. The patient and control groups had no significant difference regarding age, CVS, or gender (*P* > 0.05). Also, since all images had been retrieved from the public medical centers, the participants were also matched in terms of socioeconomic status. Table 1 presents the cephalometric craniofacial characteristics, and Table 2 presents the airway volumes and cross-sectional area in the two groups. As shown, the nasopharyngeal airway volume was 4078 ± 2885.5 mm3 and 5820.4 ± 2483.7 mm^3^ in the CLP group and the control group, respectively. Therefore, the nasopharyngeal airway volume in the CLP group was 42% smaller than that in the control group; however, this difference was not statistically significant (*P* = 0.4). The oropharyngeal airway volume was 11,165.1 ± 5553.2 mm^3^ and 16,103.7 ± 6757 mm^3^ in the CLP group and the control group, respectively. Thus, the oropharyngeal airway volume in the CLP group was 44% smaller than that in the control group, and this difference was statistically significant (*P* < 0.002). The total airway volume was 15,242.1 ± 7409.1 mm^3^ and 21,924.1 ± 8210.9 mm^3^ in the CLP group and the control group, respectively. Hence, the total airway volume in the CLP group was 43% smaller than that in the control group, and this difference was also statistically significant (P < 0.002).

**Table 1:** Cephalometric craniofacial characteristics of CLP patients and non-cleft controls

Variables	SNA angle	SNB angle	ANB angle	UP1-SN angle	SN-GoGn angle
CLP group (*n* = 30)	80.2333	79.6000	0.6333	102.8000	31.6333
Control group (*n* = 30)	81.8667	79.9333	1.9333	101.8667	31.7667
*P* value	0.001	0.374	0.022	0.078	0.518

**Table 2:** Airway volumes and cross-sectional area in CLP patients and non-cleft controls

Variables	Nasopharyngeal airway volume (mm^3^)	Oropharyngeal airway volume (mm^3^)	Total airway volume (mm^3^)	Posterior airway length (mm)	MinAx (mm^2^)
Control group (*n* = 30)	5820.4 ± 2483.7	16,103.7 ± 6757	21,924.1 ± 8210.9	13.3 ± 2.8	250.8 ± 97.2
CLP group (*n* = 30)	4078 ± 2885.5	11,165.1 ± 5553	15,242.1 ± 7409.1	5.44 ± 2.7	123.5 ± 59.6
*P* value	*P* = 0.4	*P* < 0.002	*P* < 0.002	*P* < 0.000	*P* < 0.000

The minAx was 123.5 ± 59.6 mm^2^ and 250.8 ± 97.29 mm^2^ in the CLP group and the control group, respectively. Therefore, the minAx in the CLP group was 103% smaller than that in the control group, and this difference was statistically significant (*P* < 0.000).

The PAL was 5.44 ± 2.7 mm and 13.3 ± 2.8 mm in the CLP group and the control group, respectively. Thus, the PAL in the CLP group was 144% smaller than that in the control group, and this difference was statistically significant as well (*P* < 0.000).

## Discussion

This study assessed the pharyngeal airway dimensions in non-syndromic unilateral CLP patients between 9 and 12 years, and age- and sex-matched controls using CBCT. The results showed that the oropharyngeal and the total airway volumes, the minAx, and the PAL in patients with unilateral CLP were significantly smaller than the corresponding values in the control group. Although the nasopharyngeal airway volume in CLP patients was smaller than that in the control group by 42%, this difference did not reach statistical significance probably due to high standard deviation of the data.

The current results were in agreement with the findings of Celikoglu et al. [20, 21]. In their first study, Celikoglu et al. [21] compared airway volumes of patients with unilateral CLP with a control group. In their second study [20], they compared bilateral CLP patients with controls. They obtained almost similar results in both studies that agreed with our findings, which can be attributed to relatively similar methodologies. Celikoglu et al., in one of their studies [21], used another posterior landmark instead of the posterior nasal spine (PNS), which was independent of the location of the PNS or other anatomical landmarks, and could be easily constructed and had high reproducibility. This method was later used by some other researchers and was also employed in the present study. Nonetheless, the present study was superior to those of Celikoglu et al. [20, 21] because they evaluated older patients (12–16 years) compared with the patients evaluated in the present study. It has been well documented that growth and development of the airways occur in two age ranges of 6–9 years and 12–15 years, and there is a latent period between 9 and 12 years [34]. Moreover, CBCT is not often requested for children younger than 9 years. (It is often requested prior to grafting for eruption of permanent canine teeth.) Thus, CBCT scans of 6- to 9-year-olds are difficult to find. Thus, the age range of 9–12 years is the most suitable for such studies since soft tissue changes during this period are not as noticeable as those occurring between 12 and 15 years. Thus, it may be concluded that Celikoglu et al. [20, 21] evaluated a less stable age range. Also, they performed imaging in supine position which can affect the airway dimensions. Airways are much smaller in supine position than upright position and may lead to falsely significant results [35]. Despite all the above, our results confirmed the findings of Celikoglu et al. [21]. In spite of different methodologies, landmarks, and measurements, the present results were also in agreement with the findings of Karia et al. [23] Similar to our study, they showed significantly smaller minAx in CLP patients compared with controls. The present results also agreed with those of Al-Fahdawi et al. [25, 26]. They measured the nasopharyngeal airway in their first study [25] and oropharyngeal airway in their second study [26]. They reported significantly smaller oropharyngeal airway volume in unilateral CLP patients compared with controls, but found no significant difference in the nasopharyngeal airway volume. The present results were also in accordance with the findings of Aras and Dogan [22], and Agarwal and Marwah [27] who showed smaller airway volume in CLP patients. However, Aras and Dogan [22] used 2D lateral cephalograms in their study, which have several shortcomings in comparison with CBCT [36–38]. Although Agarwal and Marwah [27] used CBCT scans, they only performed linear measurements and did not measure the volumes or cross-sectional surface areas. Shahidi et al. [29] demonstrated significantly smaller nasopharyngeal and total airway volumes in CLP patients but found no significant difference in the lower airway volume between patients and controls. Their results regarding the total airway volume were in agreement with ours. However, we reported significantly smaller oropharyngeal airway volume in CLP patients and found no significant difference in nasopharyngeal airway volume between patients and controls. This difference between the results of the two studies may be attributed to the fact that Shahidi et al. [29] evaluated patients between 17 and 45 years and did not include history of orthodontic treatment in their exclusion criteria. History of orthodontic treatment (whether functional or expansion) can serve as a confounding factor and significantly affect the results since it has been demonstrated that maxillary expansion can change the airway volume [39–42]. Moreover, they used the PNS landmark, which has a high percentage of error in CLP patients [43].

Ivy Kiemle et al. [24] measured the nasopharyngeal, oropharyngeal, and hypopharyngeal airway volumes, and the minAx in patients with unilateral CLP. They found significantly smaller mean pharyngeal airway volume in CLP patients, compared with controls, which was similar to our findings; but they did not detect any significant difference in the minAx, which was different from our finding in this respect. It should be noted that the minAx in CLP patients in their study was 30% smaller than that in the control group; however, due to high standard deviation values, this difference did not reach statistical significance. Their study had some differences with ours. Both the patient and control groups were significantly older (28.4 ± 8 and 23.2 ± 4, respectively) than the two groups in our study. Also, high standard deviation values of the mean age in both groups resulted in heterogeneity of the data. Pimenta et al. [30] found no significant difference between CLP patients and controls regarding the nasopharyngeal and oropharyngeal airway volumes. Their results cannot be well compared with ours since they used different landmarks and measurements. Moreover, patients in their study were between 7 and 12 years. As mentioned earlier, combining patients younger than 9 years with those older than 9 years of age decreases the internal reliability of the results.

Large sample size, assessment of patients between 9 and 12 years, and homogeneity of the patients and controls regarding CVS (II and III) were among the strengths of this study. Also, we used a more reliable method than using the PNS landmark. Last but not least, we measured the minAx in addition to airway volumes, which is a more important index than the total pharyngeal volume.

This study, as well as many of the above-mentioned studies, showed smaller airway volumes in prepubertal unilateral CLP patients, which may be due to surgical interventions performed for cleft closure and subsequent surgical scarring, resulting in a reduction in airway volume and cross-sectional area in these patients. Growth-related parameters may be another reason for this finding, as explained in the counterpart theory described by Enlow and Hans [44]. According to this theory, growth activity in one region may be associated with compensatory growth in other areas. Thus, anterior displacement of the nasomaxillary complex during growth and development provides adequate space for the development of the nasopharynx. Therefore, it may be concluded that anatomical discrepancies of the nasomaxillary complex in CLP patients lead to incomplete development of the pharynx, resulting in functional respiratory challenges.

Orthodontists are actively involved in diagnosis and treatment planning of CLP patients during the prepubertal and pubertal stages. Thus, they can detect and monitor the functional challenges of these patients. Growth and development of the pharynx depend on the adjacent skeletal structures, which can be modified by orthodontic, orthopedic, and orthognathic treatments and affect the respiratory capacity of patients. Thus, detection of patients at risk of airway obstruction plays an important role in treatment planning.

Although 2D images can also be used for measurement of airway dimensions, CBCT scans are preferred for this purpose due to higher accuracy and enabling the measurement of airway cross-sectional area. This is important since it has been documented that respiratory challenge is related to the narrowest cross-section perpendicular to the air passage way [45]. However, it should be noted that despite high accuracy, CBCT has an inherent error due to the respiratory phase since evidence shows that respiration during scanning affects the dimensions and morphology of the airways [36]. Nonetheless, CBCT is still much more accurate than lateral cephalometry for such measurements.

Although our sample size was larger than that of the majority of similar previous studies, limited sample size was a limitation of this study. Also, this study had a retrospective design; thus, information about some confounding factors (having a cold or an upper airway inflammatory disease at the time of taking the CBCT scans for the control group) was not available, which was another limitation of this study. Also, long scanning time is one drawback of the currently available CBCT scanners. Thus, the patients cannot be asked to hold their breath during the scanning. As a result, breathing during scanning affects the size of the airways.

Future studies are recommended to include a control group with skeletal class III relationship due to maxillary deficiency to eliminate the effect of maxillary retrognathism as a confounder, and provide more accurate results. Moreover, to our knowledge, all studies available on this topic have a retrospective design. A prospective study, according to ethical guidelines, is required to re-assess the patients after alveolar grafting and analyze the effects of grafting on airway dimensions.

## Conclusions

- Oropharyngeal airway dimension of non-syndromic unilateral CLP patients was smaller than non-cleft controls.
- The total airway volumes and posterior airway length in non-cleft controls were larger than the non-syndromic unilateral CLP patients.
- The minimal cross-sectional area of the airway in non-syndromic unilateral CLP patients was smaller than non-cleft controls.

## Data Availability

The datasets used and/or analyzed during the current study are available from the corresponding author on reasonable request.

## Abbreviations

CLP: Cleft lip and palate
CBCT: Cone-beam computed tomography
3D: Three-dimensional
minAxa: Minimal cross-sectional area of the airway
PAL: Posterior airway length
CVS: Cervical vertebral stage
